# Satellite DNA Mapping in *Pseudis fusca* (Hylidae, Pseudinae) Provides New Insights into Sex Chromosome Evolution in Paradoxical Frogs

**DOI:** 10.3390/genes10020160

**Published:** 2019-02-19

**Authors:** Kaleb Pretto Gatto, Karin Regina Seger, Paulo Christiano de Anchieta Garcia, Luciana Bolsoni Lourenço

**Affiliations:** 1 Laboratory of Chromosome Studies, Department of Structural and Functional Biology, Institute of Biology, University of Campinas, Campinas 13083-863, SP, Brazil; karinsbio@yahoo.com.br; 2 Department of Zoology, Institute of Biological Sciences, Federal University of Minas Gerais, Belo Horizonte 31270-901, MG, Brazil; pcagarcia@gmail.com

**Keywords:** chromosome evolution, chromosome rearrangements, phylogeny, satellite DNA, sex chromosomes

## Abstract

In the frog genus *Pseudis*, previous works found a sex-linked heteromorphism of the PcP190 satellite DNA in the nucleolus organizer region (NOR)-bearing chromosome pairs of *Pseudis bolbodactyla* and *Pseudis tocantins*, which possess a ZZ/ZW sex determination system. A pericentromeric inversion was inferred to have occurred during W chromosome evolution, moving a chromosomal cluster enriched by the PcP190 from the short arm (as observed in *P. bolbodactyla*) to the NOR-bearing long arm (as observed in *P. tocantins*). However, whether such an inversion happened in *P. tocantins* or in the common ancestor of *Pseudis fusca* and *P. tocantins* remained unclear. To assess this question, we mapped PcP190 in the karyotype of *P. fusca* from three distinct localities. Southern blotting was used to compare males and females. The mitochondrial H1 fragment (which contains the 12S ribosomal RNA (rRNA), tRNAval, and 16S rRNA genes) and cytochrome b gene were partially sequenced, and a species tree was inferred to guide our analysis. *Pseudis fusca* specimens were placed together as the sister group of *P. tocantins*, but based on genetic distance, one of the analyzed populations is probably an undescribed species. A cluster of PcP190, located in the long arm of chromosome 7, is sex linked in this putative new species but not in the remaining *P. fusca*. We could infer that the pericentromeric inversion that moved the PcP190 site to the NOR-bearing chromosome arm (long arm) occurred in the common ancestor of *P. fusca*, the putative undescribed species, and *P. tocantins*.

## 1. Introduction

Sex chromosomes originate from an autosomal pair after the acquisition of a sex determination locus (reviewed in [1,2]). During sex chromosome evolution, sex-specific gene accumulate in the sex-determining region and suppression of the recombination between the sex chromosomes occurs. Because of this recombination suppression, chromosome Y or W may undergo degeneration, which involves the loss of genes and the accumulation of repetitive DNA, leading to highly heteromorphic sex chromosomes (reviewed in [3,4,5]).

Among anurans, only 52 [6,7,8] karyotyped species show heteromorphic sex chromosomes, while most species possess homomorphic sex chromosomes, which are not differentiated by classical cytogenetic techniques. In this order of amphibians, an interesting case is observed in the genus *Pseudis*, in which species with different levels of sex chromosome differentiation are present. In *Pseudis tocantins*, the W chromosome is easily differentiated from the Z chromosome by chromosomal size and the locations of the nucleolus organizer region (NOR) and heterochromatin sites [9]. The W chromosome of this species is submetacentric and larger than the Z chromosome due to accumulation and amplification of the heterochromatin in the long arm (Wq), which is enriched by the satellite DNA (satDNA) PcP190 [9,10]. In *Pseudis bolbodactyla*, sex chromosome heteromorphism can be detected only by the presence of a single cluster of PcP190 satDNA in the short arm of the W chromosome, which is absent in the Z chromosome, although these chromosomes do not differ in size [11]. However, in *Pseudis paradoxa*, *Pseudis minuta*, and *Pseudis cardosoi*, no sex chromosome heteromorphism has been identified, and the clusters of PcP190 satDNA are not sex-linked. Chromosome mapping of PcP190 sequences revealed a cluster in chromosome 7 of male and female *P. paradoxa* and *P. minuta*, and in chromosome 5 of male and female *P. cardosoi*, which are homeologous to the sex chromosomes of *P. bolbodactyla* and *P. tocantins* [11].

PcP190 satDNA is considered to be derived from 5S ribosomal DNA (rDNA) [12] and is widespread in the frog superfamily Hyloidea, since it has been previously reported for the leptodactylid genera *Engystomops*, *Leptodactylus*, and *Physalaemus*, the hylodid genus *Crossodactylus* and the hylid genera *Lysapsus* and *Pseudis* (reviewed in [6,11]). Chromosomal clusters of PcP190 were detected under sex-linked conditions only in *Pseudis tocantins* and *Pseudis bolbodactyla*, although the PcP190 clusters detected in the Z and W chromosomes in *Physalaemus ephippifer* differed in size, with the largest cluster present in the W chromosome [13]. Sequence analyses have shown the existence of a more conserved region (CR) in PcP190 repeats, which shows high similarity among all species and corresponds to the 5S ribosomal RNA (rRNA) gene, and a hypervariable region (HR) that enables the identification of distinct sequence groups [10,11,13]. In the genus *Pseudis*, eight different PcP190 groups (PcP-1 to PcP-7 and PcP-9) were previously described [11].

Based on PcP190 satDNA mapping, along with the classical cytogenetic data generated by Busin et al. [9], Gatto et al. [11] proposed the occurrence of a pericentromeric inversion that carried the PcP190 site from the short arm (as observed in *P. minuta*, *P. cardosoi*, *P. bolbodactyla*, and *P. paradoxa*) towards the long arm, which also bears the NOR sites (as observed in *P. tocantins*). However, because of PcP190 mapping in the karyotype of *P. fusca*, the sister taxon of *P. tocantins* was not available, and the authors could not infer whether this event occurred in the common ancestor of *P. tocantins* and *P. fusca* or in the lineage that gave rise to *P. tocantins*.

In the present work, we analyzed the chromosomal localization of PcP190 satDNA clusters in *Pseudis fusca*, aiming to test the hypothesis that the abovementioned pericentromeric inversion occurred in the common ancestor of *P. tocantins* and *P. fusca*. We included the cytogenetically studied individuals in a phylogenetic analysis along with other species of the genus *Pseudis* to map the obtained chromosome characters on the generated species tree.

## 2. Material and Methods

### 2.1. Cytogenetic Analysis

#### 2.1.1. Individuals, Chromosome Preparations, and Karyotype Description

For the cytogenetic study, we analyzed eight males of *Pseudis fusca* from Coronel Murta–MG (ZUEC 13235, ZUEC 13237 ZUEC 13239, and MNRJ 35459–35461), a male and a female of *P. fusca* from Salinas–MG (ZUEC 24517 and ZUEC 24518), and three males and six females from Carlos Chagas–MG (ZUEC 22076–22079, UFMG-A 17455, 17457, 17468, 17469, and 15136).

The chromosome preparations of *Pseudis fusca* from Coronel Murta and Salinas were obtained from suspensions of intestinal epithelial cells made available by Busin et al. [9] at the Laboratory of Chromosome Studies (LabEsC) of the University of Campinas. The chromosome preparations of *P. fusca* from Carlos Chagas were obtained from the intestinal epithelial cells of animals previously treated with 1% colchicine solution following the method described by King and Rofe [14] with modifications from Gatto et al. [11]. This protocol was approved by the Committee for Ethics in Animal Use of the University of Campinas (CEUA/UNICAMP) (protocol #3419-1). All individuals from Carlos Chagas were collected under authorization from Instituto Chico Mendes de Conservação da Biodiversidade and Sistema de Autorização e Informação em Biodiversidade (ICMBio/SISBIO) (authorization #45183-3).

Chromosome preparations from the specimens from Carlos Chagas were subjected to conventional staining with 10% Giemsa solution, C-banding according to Sumner [15], and silver impregnation by the Ag-NOR method following Howell and Black [16]. The metaphases subjected to C-banding were sequentially stained with the base-specific fluorochromes 4′,6-diamidino-2-phenylindole (DAPI; at a final concentration of 0.5 µg/mL) and mithramycin (MM; at a final concentration of 0.5 mg/mL) after the removal of Giemsa using 70% ethanol. These classical cytogenetic techniques allowed for karyotype description of the specimens from Carlos Chagas, since these specimens had not been karyotyped previously.

#### 2.1.2. Amplification of PcP190 Satellite DNA for Further Use as a Probe in Southern Blotting and In Situ Hybridization

To obtain PcP190 satDNA probes, PcP190 sequences were isolated from the genomic DNA of *Pseudis fusca*. Samples of genomic DNA were obtained from liver tissue of a male and a female of *P. fusca* (Table S1), according to Medeiros et al. [17]. For PCR amplification of PcP190 satDNA, the P190F and P190R primers were used [12]. The PCR products were purified using the Wizard SV Gel and PCR Clean-up System (Promega, Madison, WI, USA). The obtained fragments were inserted into a plasmid using the pGEM-T Easy Vector Kit (Promega, USA) and cloned into *Escherichia coli* JM109 employing the TransformAid Bacterial Transformation Kit (Fermentas, Waltham, MA, USA), according to the manufacturer’s instructions. Recombinant colonies were selected, and the plasmids were extracted according to Sambrook et al. [18]. The inserts were sequenced using the BigDye Terminator Kit (Applied Biosystems, Waltham, MA, USA) after being PCR-amplified with T7 and SP6 universal primers. Nucleotide sequences were obtained from an ABI PRISM 3130 XL Genetic Analyzer (Hitachi, Tokyo, Japan) by the Facility of DNA Sequencing of the Chemistry Institute of the University of São Paulo and were deposited in the GenBank under accession numbers MH571141–MH571149. The treatment of repetitive monomers for sequence alignment, recognition of more conserved and hypervariable regions, and classification of the cloned PcP190 fragments followed the proposal of Gatto et al. [10]. A similarity analysis of more conserved and hypervariable regions was performed in BioEdit [19].

#### 2.1.3. Southern Blot Detection of PcP190 Satellite DNA

Since there are differential amounts of PcP190 satDNA related to sex in *Pseudis bolbodactyla* and *P. tocantins* [10,11], Southern blot experiments were performed to evaluate the presence and organization of PcP190 in eleven males and seven females of *Pseudis fusca* (see Table S1 for details). Genomic DNA was extracted by the standard phenol:chloroform method [18] and digested with *Stu*I or HindIII restriction endonuclease, according to Gatto et al. [11]. The restriction fragments were subjected to electrophoresis in a 1.2% agarose gel and then transferred to a nitrocellulose membrane, according to Sambrook et al. [18]. The membrane containing the restriction fragments was hybridized with probes for PcP-2 sequences, since the PcP190 sequences isolated from *P. fusca* genomic DNA by PCR were classified as belonging to the PcP-2 type of PcP190 satDNA (see Results). Probe labeling, hybridization, and detection followed Gatto et al. [11].

#### 2.1.4. Fluorescent In Situ Hybridization and Comparative Genomic Hybridization

To map PcP190 satDNA sequences in the karyotypes in the study, probes for PcP-2 sequences were obtained by PCR amplification of a fragment isolated from *Pseudis fusca* (PcP190-2-Pfus-M-C3) using the PCR Dig Probe Synthesis Kit (Roche, Penzberg, Germany). The probes were ethanol-precipitated in the presence of salmon sperm DNA (100 ng/µL) and resuspended in hybridization buffer (50% formamide, 2× SSC and 10% dextran sulfate). The probe hybridization and washing steps followed Viegas-Péquignot [20]. For probe detection, chromosome preparations were incubated with rhodamine-conjugated anti-digoxigenin (600 ng/µL) for 45 min, washed with PBT solution (1× PBS, 0.4% BSA and 0.1% Tween 20) and counterstained with 0.5 µg/mL DAPI. Individuals used for fluorescent in situ hybridization (FISH) experiments are indicated in Table S1.

As the sex-specific PcP190 chromosomal site was observed in *Pseudis fusca* from Carlos Chagas (see Results section), we also employed comparative genomic hybridization (CGH) to assess the presence of sex chromosome heteromorphism in this case. Female genomic DNA (1 µg) of *P.* fusca from Carlos Chagas was labeled with FITC-12-dUTP (Roche, Germany) by a Nick Translation Kit (Roche, Germany). The probe was ethanol-precipitated and resuspended in hybridization buffer (50% formamide, 2× SSC and 10% dextran sulfate). A probe mix was made with competitor DNA obtained from genomic DNA from a male of *P. fusca* from Carlos Chagas, following Gatto et al. [10], in a proportion of 1:10 (probe:competitor DNA). The probe hybridization and washing steps followed Viegas-Péquignot [20]. Chromosome preparations were counterstained with 0.5 µg/mL DAPI.

Chromosome preparations were observed with an Olympus BX-60 (Olympus, Tokyo, Japan) fluorescence microscope. Images were captured with a Q-Color 3 (Olympus, Japan) digital system and edited using only the brightness and contrast options in Adobe Photoshop CS3 (Adobe Systems, San Jose, CA, USA).

### 2.2. Phylogenetic and Genetic Distance Analyses

Specimens from all sites sampled for cytogenetic analysis were included in a phylogenetic analysis to aid in the evolutionary interpretation of the chromosomal data. As the specimens from Carlos Chagas showed considerable differences with respect to PcP190 satDNA amounts and chromosomal mapping when compared with the remaining specimens of *Pseudis fusca* (see Results section for details), we also conducted an analysis of genetic distance based on mitochondrial nucleotide sequences, as described below.

#### 2.2.1. Taxon and Gene Sampling

Nucleotide sequences of the mitochondrial H1 fragment (which includes the 12S rRNA, tRNAval and 16S rRNA genes) and cytochrome b gene (cytb) were generated for specimens of *Pseudis fusca* from Coronel Murta–MG, Salinas–MG, and Carlos Chagas–MG (Table S2). Nucleotide sequences from *Pseudis* available in GenBank were also included (Table S2). Those sequences identified in GenBank as belonging to *P. platensis* were considered representative of *P. paradoxa* based on discussions provided by Garda et al. [21] and Santana et al. [22]. As an outgroup, we used the four species of the genus *Lysapsus*, since this genus was inferred as the sister group of *Pseudis* by Wiens et al. [23] and Duellman et al. [24]. For genetic distance analyses, two sequences of *P. fusca* from Jequitinhonha–MG, containing only the 16Sar-16Sbr fragment (a partial segment of the 16S rRNA gene, which is delimited by the primers 16Sar and 16Sbr [25]), and sequences of the H1 fragment produced by Garda and Cannatella [26] were also included.

#### 2.2.2. DNA Extraction and Sequencing of H1 and Cytb Fragments

Genomic DNA was extracted from liver samples following the protocol used by Medeiros et al. [17]. The H1 and cytb fragments were amplified by PCR using the Illustra Ready-To-Go PCR Beads (GE Healthcare, Chicago, IL, USA), the primers MVZ59/MVZ50 [27] and 12SL13 [28]/16Sbr [25] in the case of the H1 fragments, and the primers MVZ15 [29] and H15149(H) [30] for the amplification of the cytb fragments. The resulting amplicons were purified using the Wizard SV Gel and PCR Clean-Up System (Promega, USA), and sequenced with the same primers used for PCR. In the case of the amplicon obtained with the primers 12SL13 and 16Sbr, the following primers were also employed for sequencing: H1KR [31], Hedges16SL2a [32], Hedges16SH10 [32], and 16Sar [25]. Sequencing reactions were prepared using BigDye Terminator (Applied Biosystems, USA), according to the manufacturer’s instructions. Nucleotide sequences were obtained by an ABI PRISM 3130XL Genetic Analyzer automated sequencer (Hitachi, Tokyo, Japan) at the DNA Sequencing Facility of the Chemistry Institute of University of São Paulo.

#### 2.2.3. Sequence Analyses

DNA sequences were edited using BioEdit v.7.0.9.0 [19] and aligned using Muscle [33], as implemented in Analysis Tool Web Services [34]. A matrix containing 36 operational taxonomic unit (OTUs) and 2724 characters of the H1 and cytb fragments was obtained and used for inferring phylogenetic relationships using Bayesian analysis in MrBayes v.3.2.6 [35]. The evolutionary model SYM+G was inferred using Mr. Modeltest [36] for both H1 and cytb partitions. A Bayesian analysis was performed using 10 million generations and sampling one tree every 100 generations. Consensus topology with posterior probability for each node was obtained after discarding the first 25% of the trees generated.

As the 16S rRNA gene has been considered a helpful marker for studies between candidate species of anurans [37,38], two different matrices were used for the genetic distance analyses in *Pseudis*: (a) a matrix with 484 bp (corresponding to the fragment of the 16S rRNA gene delimited by the primers 16Sar and 16Sbr) and 38 OTUs, including sequences of *Pseudis fusca* that are not present in the phylogenetic analysis (Jequitinhonha–MG) (Table S2), and (b) a 2340 bp matrix with the H1 fragments of 36 OTUs. Genetic distances (p-distance) were calculated using MEGA 7.0 [39], not considering gaps in pairwise comparisons.

## 3. Results

### 3.1. Classical Cytogenetic Analysis of *Pseudis fusca* from Carlos Chagas–MG

**Figure 1:**
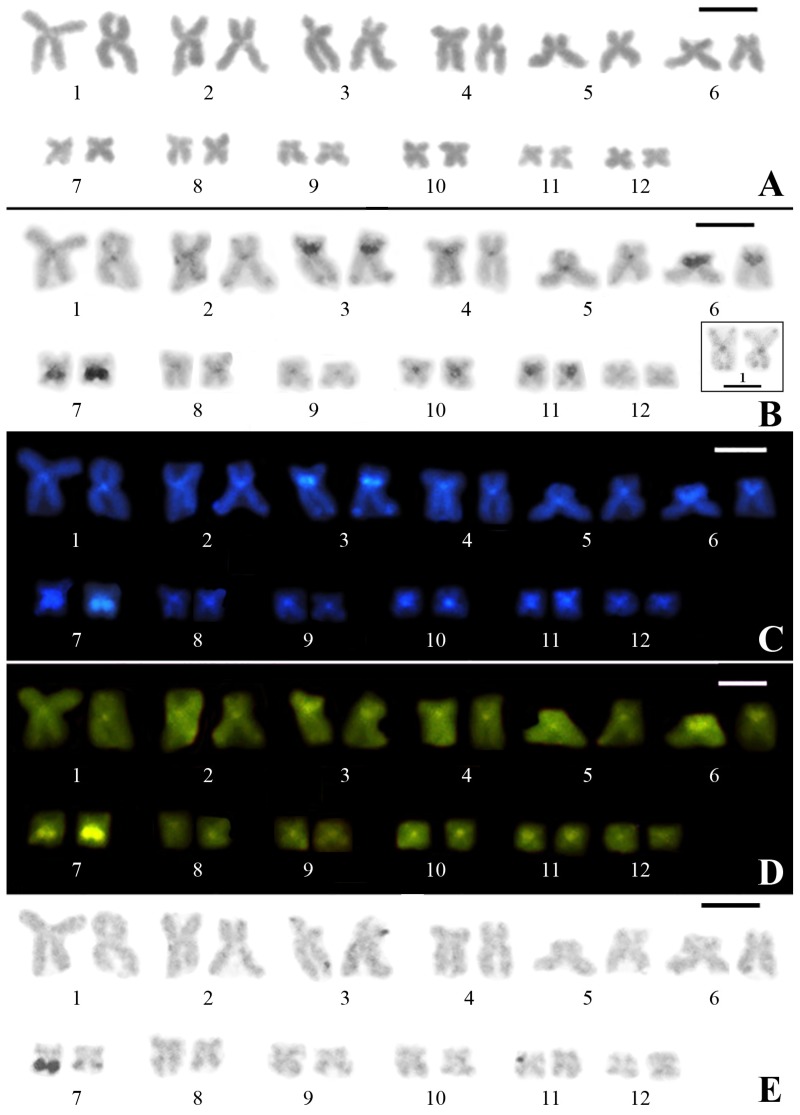
Karyotype of a female of *Pseudis fusca* from Carlos Chagas–MG subjected to Giemsa conventional staining (**A**), C-banding (**B**), C-banding followed by DAPI (**C**), and MM staining (**D**), and silver impregnation by the Ag-NOR method (**E**). The inset in (**B**) shows chromosome pair 1 obtained from another metaphase, in which the terminal C-bands clearly can be seen. Bar: 5 µm.

This karyotype is composed of 24 chromosomes, with six metacentric chromosome pairs (pairs 1, 2, 7, 8, 11 and 12), five submetacentric (pairs 3, 4, 5, 9 and 10), and one subtelocentric (pair 6) (Figure 1). All of the chromosomes present centromeric heterochromatin, and pericentromeric heterochromatic blocks can be observed in the short arms of chromosomes 3 and 6 and in the long arm of chromosome 7. Faint terminal blocks of heterochromatin are also observed in chromosomes 1, 2, and 3, although they are hardly seen in some metaphases, especially those of chromosome 1 (Figure 1).

In the chromosome preparations sequentially subjected to C-banding and staining with fluorochromes, all centromeric regions showed DAPI- and MM-positive staining (Figure 1C,D). However, the heterochromatic blocks in the terminal regions of chromosomes 2 and 3 showed exclusively DAPI-positive staining. C-bands stained with both fluorochromes were also present pericentromerically in the short arms of chromosomes 3 and 6 and the long arm of chromosome 7.

**Figure 2:**
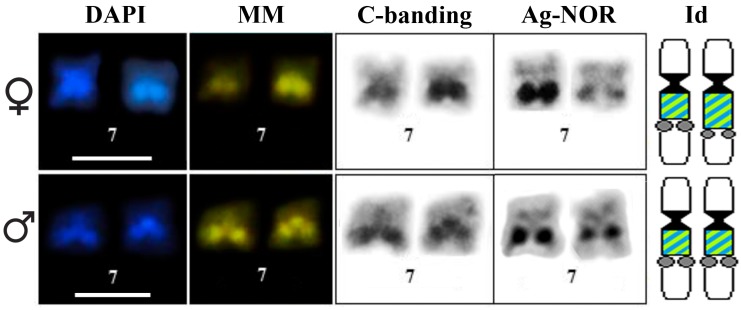
Pair 7 of the female ZUEC 22078 (upper panel) and the male ZUEC 22077 (bottom panel) of *Pseudis fusca* from Carlos Chagas–MG sequentially subjected to C-banding, DAPI, and MM staining and silver impregnation (Ag-NOR method). In the ideograms (Id) of pair 7, NORs are indicated by gray circles, centromeres are in black, and adjacent to them is the heterochromatin in green and blue, representing simultaneous DAPI/MM staining. Note that the centromeres are also stained with DAPI and MM, but this is not shown in the ideograms (Id). Bar: 5 µm.

The NOR sites are located adjacent to the pericentromeric heterochromatic block in the long arm of chromosome 7 (Figure 2). The three individuals analyzed showed NOR size heteromorphism. Furthermore, the female individual (ZUEC 22078) showed size heteromorphism of the heterochromatin in pair 7, with the homologue that bears a small NOR presenting a large heterochromatic block that was strongly stained with both fluorochromes (Figure 2).

### 3.2. PcP190 satellite DNA and Comparative Genomic Hybridization

**Figure 3:**
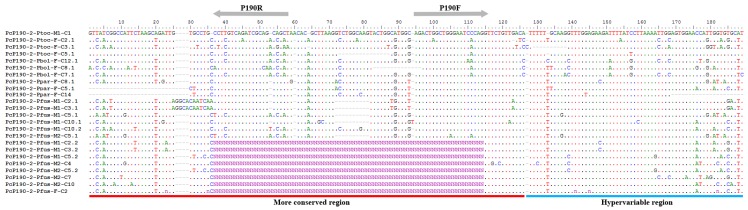
Alignment of the PcP190 satDNA sequences isolated from *Pseudis fusca* with complete sequences from the PcP-2 group previously isolated from *P. tocantins* (KX170921, KX170922, KX170923, KX170924), *P. bolbodactyla* (MH370391, MH370392, MH370395), and *P. paradoxa* (MH370405 and MH370407). Gray arrows indicate the P190F and P190R primer annealing regions. Sequences that show the central part of the CR with N breaks represent partial monomers.

A total of nine clones were obtained from male (from Coronel Murta–MG) and female (from Salinas–MG) genomic DNA of *Pseudis fusca*. Six of the cloned fragments exhibited more than one repeat unit, while the remaining four clones had a partial monomer only. Based on the HR, all the sequences were identified as belonging to the PcP-2 group (Figure 3). The similarity of the HRs of the sequences from *P. fusca* to the other PcP-2 sequences previously isolated from *Pseudis* species was 83.82% (see Figure S1 for details on the compared sequences). With respect to the CR of this satDNA, the average similarity of the sequences from *P. fusca* to all previously described PcP190 sequences was 75.31%, and that to the other PcP-2 sequences was 75.24% (only complete CRs were considered for this analysis—see Figure S1). Two sequences obtained from *P. fusca* (PcP190-2-Pfus-M1-C2.1 and PcP190-2-Pfus-M1-C2.1 3.1) presented the insertion of a 12-bp segment in the CR that was not observed in the other Pcp-2 sequences (Figure 3). When these sequences were excluded from the analysis, the similarity values between the sequences from *P. fusca* and the other sequences of PcP190 and the PcP-2 sequence group were 78.13% and 78.62%, respectively.

When all 16 complete CRs from the PcP-2 group sequenced to date for the genus *Pseudis* (shown in Figure 3) were compared with each other, the mean similarity observed was 77.06%. Similarly, the mean similarity of all 45 HRs from the PcP-2 group described to date was 83.39% (see Figure S1 for details on the compared sequences).

**Figure 4:**
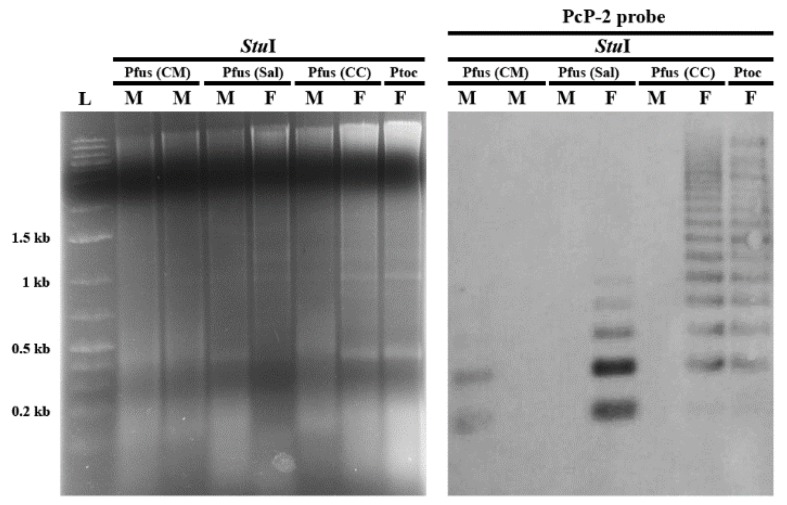
Representative Southern blot analysis of PcP-2 sequences from two males (M) of *Pseudis fusca* from Coronel Murta–MG (Pfus (CM)), a male (M) and a female (F) of *P. fusca* from Salinas–MG (Pfus (Sal)), a male and a female of *P. fusca* from Carlos Chagas–MG (Pfus (CC)), and a female of *P. tocantins* as a positive control (Ptoc).

**Figure 5:**
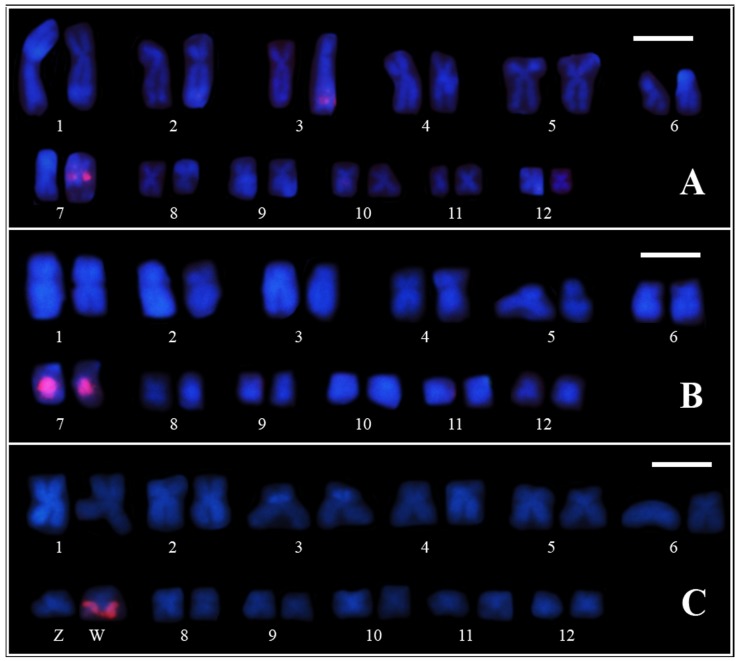
Fluorescent in situ hybridization of PcP190 satDNA in a male karyotype of *Pseudis fusca* from Coronel Murta–MG (ZUEC 13235) (**A**), a female karyotype of *P. fusca* from Salinas–MG (ZUEC 24518) (**B**), and a female of *P. fusca* from Carlos Chagas–MG (ZUEC 22078) (**C**). Bar: 5 µm.

Southern blot analysis of PcP-2 sequences in *Pseudis fusca* from Coronel Murta–MG and Salinas–MG populations indicated an intraspecific variation in PcP190 satDNA, but this variation was not sex related (Figure 4 and Table S1). Among the seven analyzed males of *P. fusca* from Coronel Murta–MG, only one individual (ZUEC 13235) showed a ladder pattern of hybridization. Among the specimens from Salinas–MG, the female individual (ZUEC 24518) also showed the ladder pattern of hybridization typical of satDNA, while the male individual (ZUEC 24517) did not show any hybridization signal. In the karyotype of the male ZUEC 13235 from Coronel Murta–MG, FISH analysis with PcP-2 probes detected a single hybridization signal in the pericentromeric heterochromatic region of the long arm in one of the homologues of pair 7 (Figure 5A). In contrast, the karyotype of the female ZUEC 24518 from Salinas–MG showed a strong hybridization signal from the same probe in the heterochromatin of the long arms of both homologues of pair 7 (Figure 5B). Chromosome mapping of the PcP-2 sequences in the male MNRJ 35460 from Coronel Murta–MG, which was not subjected to Southern blot analysis, showed the same result as that obtained for the male ZUEC 13235 (see a summary of these results in Table S1).

In contrast to *Pseudis fusca* from Coronel Murta and Salinas, the Southern blot experiment showed a sex-related variation for *P. fusca* from Carlos Chagas–MG. The six analyzed females from this latter locality showed a ladder pattern for PcP-2 sequences in the Southern blots, whereas the three males from this site showed no signal in this experiment (Figure 4 and Table S1). FISH assays with PcP-2 sequences as probes in the female karyotype of *P. fusca* from Carlos Chagas–MG showed a single hybridization signal in the pericentromeric heterochromatin in the long arm of one of the homologues of pair 7 (Figure 5C), while no signal was observed with the PcP-2 probes in the male karyotypes of this population.

**Figure 6:**
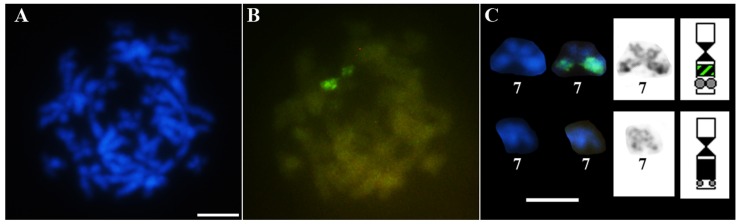
Comparative genomic hybridization in *Pseudis fusca* from Carlos Chagas–MG. Chromosome preparation stained with DAPI (**A**) and subjected to CGH (**B**). (**C**) Chromosome pair 7 from the metaphase shown in (**A**,**B**) stained with DAPI, subjected to CGH, and silver-impregnated by the Ag-NOR method. In the ideograms, the heterochromatic regions are shown in black, the NORs in gray circles, and the region revealed by CGH in dashed green. Bar: 5 µm.

CGH experiments in *Pseudis fusca* from Carlos Chagas–MG showed a strong hybridization signal with the female genomic DNA probe in one of the homologues of pair 7, in the same region where the PcP190 satDNA was mapped by FISH (Figure 6).

### 3.3. Phylogenetic Inferences and Genetic Distances

**Figure 7:**
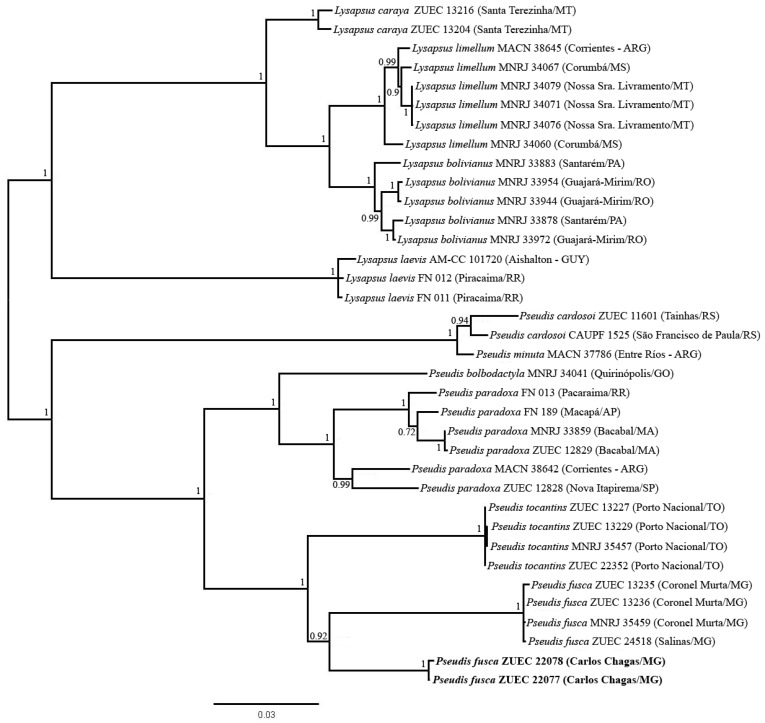
Phylogenetic relationships of *Pseudis* and *Lysapsus* inferred by Bayesian analysis of the mitochondrial H1 and cytb fragments. Numbers at the nodes indicate posterior probability values. *Pseudis fusca* from Carlos Chagas – MG is in bold.

In the Bayesian inference, all specimens of *Pseudis fusca* were grouped together in the same clade, which had a high posterior probability and was inferred as the sister group of *P. tocantins* (Figure 7). Based on either the 16S rDNA segment or the H1 fragment, high divergence was estimated between *P. fusca* from Carlos Chagas–MG and the other specimens of *P. fusca*. High genetic distance was also found between *P. fusca* from Carlos Chagas–MG and *P. tocantins* (Table 1). In contrast, among the specimens of *P. fusca* from Carlos Chagas–MG, no genetic distance was observed for the 16Sar-16Sbr fragment of the 16S rRNA gene and only 0.17% of genetic distance was calculated for the H1 fragment. Similarly, among the remaining specimens of *P. fusca* (i.e., specimens from Coronel Murta/Salinas/Araçuaí/Jequitinhonha), no divergence was observed in the 16Sar-16Sbr fragment, and only 0.13% of genetic distance was estimated from the H1 fragment (Table 1—diagonal line).

## 4. Discussion

### 4.1. Genetic Divergence: Could *Pseudis fusca* from Carlos Chagas be a New Species?

Previous studies involving phylogenetic inferences with molecular data showed *Pseudis fusca* as the sister taxon of *P. tocantins* [23,24,26,40]. In the present work, the specimens of *P. fusca* also composed the sister group of *P. tocantins*, but high genetic divergence was found inside the *P. fusca* clade. The individuals of *P. fusca* from Carlos Chagas–MG highly diverged from the remaining specimens of this species, which came from four different localities and were very similar in the H1 fragment (99.87% similar in the H1 fragment, with no divergence present in the 16Sar-16Sbr fragment). The genetic distances estimated between *P. fusca* from Carlos Chagas–MG and the group composed of all other *P. fusca* specimens were high for both the 16Sar-16Sbr fragment (>7%) and the H1 fragment (5.98%). These estimated divergence values are similar to those found between other species of *Pseudis* and are higher than those observed between *P. bolbodactyla* and *P. paradoxa* (6.07% and 5.66% for the 16Sar-16Sbr fragment and H1 fragment, respectively), and between *P. minuta* and *P. cardosoi* (1.25% and 1.47% for the 16S gene fragment and H1 fragment, respectively) (Table S2).

DNA sequences have been highly important in species delimitation studies, contributing in some cases to the detection of undescribed species [41,42,43,44]. For anurans, the 16S mitochondrial rRNA gene has been proposed as a good marker for DNA barcoding studies [37,38,45], since it shows low intraspecific genetic divergence and high interspecific genetic distances [37]. Fouquet et al. [38] proposed that a genetic distance higher than 3% in the 16Sar-16Sbr fragment could be enough to suggest the presence of unnamed species in the studied sample, and using this approach, the authors identified 129 candidate species among neotropical anurans. Although this threshold should be taken with caution, since several species have been recognized with lower genetic distance values [46,47], it has been helpful in several cases. The genetic divergences estimated here between the Carlos Chagas–MG population and the other specimens of *Pseudis fusca* from both the 16Sar-16Sbr fragment and the H1 fragment are much higher than 3%, and may indicate that the individuals from Carlos Chagas–MG represent an undescribed species. Although for a proper taxonomic revision of *P. fusca*, a larger sampling from Carlos Chagas – MG and other *P. fusca* populations is still needed, as well as morphological and acoustical analyses for clarity when discussing the evolutionary chromosomal changes in the genus *Pseudis*; in Section 4.3 we used *Pseudis* sp. to refer to the population from Carlos Chagas–MG.

### 4.2. PcP190 Sequences of *Pseudis fusca*

Distinct types of PcP190 satDNA have been described based on a hypervariable region (HR), which is juxtaposed to a more conserved region (CR) composed of approximately 120 bp [10,11,13]. The variation (in size and nucleotide sequences) between the distinct HR classes is so prominent that it prevents their reliable alignment [10,11]. To date, eight types of PcP190 sequences have been found in the genus *Pseudis*, with seven found in *P. tocantins*, a species that shows a conspicuous amplification of this satDNA [10]. Based on the similarity between CR and the transcribing region of 5S rDNA, Gatto et al. [10] argued that illegitimate recombination between 5S rDNA and PcP190 satDNA may play a role in the origin of new variants of HR, as they would originate from different non-transcribing sequences (NTS) of 5S rDNA [10]. This does not discard, however, the hypothesis that CR is under higher selective pressure than HR, as previously supposed by Vittorazzi et al. [16].

The PcP190 sequences obtained from *Pseudis fusca* are very similar to those classified previously as PcP-2 [10,11]. By sequencing PcP190 sequences from *P. fusca*, we increased the number of complete CR and HR sequences to 16 and 45, respectively, enabling a comparative analysis of similarity between CRs and HRs of a single type of PcP190 satDNA. As the mean similarity of the CR sequences was not higher than the mean similarity between the HR sequences, we provide here preliminary evidence against the hypothesis of differential selective pressure between CR and HR. Further analysis, however, with a larger number of sequences and studies investigating the possible role of PcP190 satDNA in heterochromatin organization is still necessary for a proper analysis of this issue.

### 4.3. Chromosome Rearrangements as a Major Force of Sex Chromosome Differentiation in *Pseudis*

The karyotype of *Pseudis* sp. presents 24 chromosomes, as previously observed for the *Pseudis* species [9,48,49], except *Pseudis cardosoi*, which has 2n = 28 because of two centric fission events [48]. By chromosome morphology, the karyotype of *Pseudis* sp. is highly similar to the 24-chromosome karyotypes of the other species of the genus *Pseudis*.

The chromosomal heterochromatin sites also seem to be conserved in *Pseudis* at some level. The distal positive C-bands in the long arms of chromosomes 2 and 3 of *Pseudis bolbodactyla*, *P. fusca*, *P. paradoxa*, and *P. tocantins* [9] also occur in *Pseudis* sp. and can be used as cytogenetic markers that suggest homeology between those chromosomes in these species. The distal heterochromatin blocks of chromosome pair 1 of *Pseudis* sp., on the other hand, were not observed in chromosome 1 of any other species of *Pseudis* [9,48]. However, these terminal C-bands are hardly seen even in metaphases from *Pseudis* sp.; thus, we could not discard the hypothesis that they are present in other *Pseudis* species but were not noticed.

Regarding NOR patterns, *Pseudis* sp. is similar to all other species of *Pseudis* with 2n = 24 [9], since in all of them the NOR is located in the long arm of chromosome 7 (which is the sex chromosome pair in *P. tocantins* [9] and *P. bolbodactyla* [11]). In *P. cardosoi*, the NOR-bearing chromosome is classified as chromosome 5, which is considered homeologous to chromosome 7 of the other species of *Pseudis* [48].

The presence of the ladder pattern of hybridization of the PcP190 satDNA probe in Southern blot experiments exclusively in females of *Pseudis* sp., the hybridization signals from a PcP190 satDNA probe in only one of the homologues of pair 7 of this species, and CGH experiments suggest that pair 7 could be the sex chromosome pair of *Pseudis* sp. from Carlos Chagas. According to this hypothesis, *Pseudis* sp. would have a ZZ/ZW sex determination system, with an incipient sex chromosome heteromorphism that could be detected only by mapping the PcP190 satDNA, similar to the condition previously observed for *P. bolbodactyla* [11]. 

Intriguingly, in *Pseudis fusca*, the sister taxon of *Pseudis* sp., no evidence of sex-linked heteromorphism in the PcP190 satDNA exists, and the same situation is observed in *P. paradoxa*, *P. minuta*, and *P. cardosoi* [11]. Although FISH and Southern blot experiments indicate intraspecific variation (i.e., a polymorphism) related to PcP190 satDNA in *P. fusca*, this variation is not sex-linked, since two different patterns were present among the nine analyzed males, and one of the patterns was the same pattern presented by a female (Table S1). In the case of *Pseudis* sp., although nine specimens were analyzed (6 females and 3 males), we cannot discard the possibility that a larger sample may show that the variation in PcP190 satDNA is not actually related to sex. However, until a larger sample is available for analysis, we will take the hypothesis that the differential amount of PcP190 satDNA may be sex-related in *Pseudis* sp.

**Figure 8:**
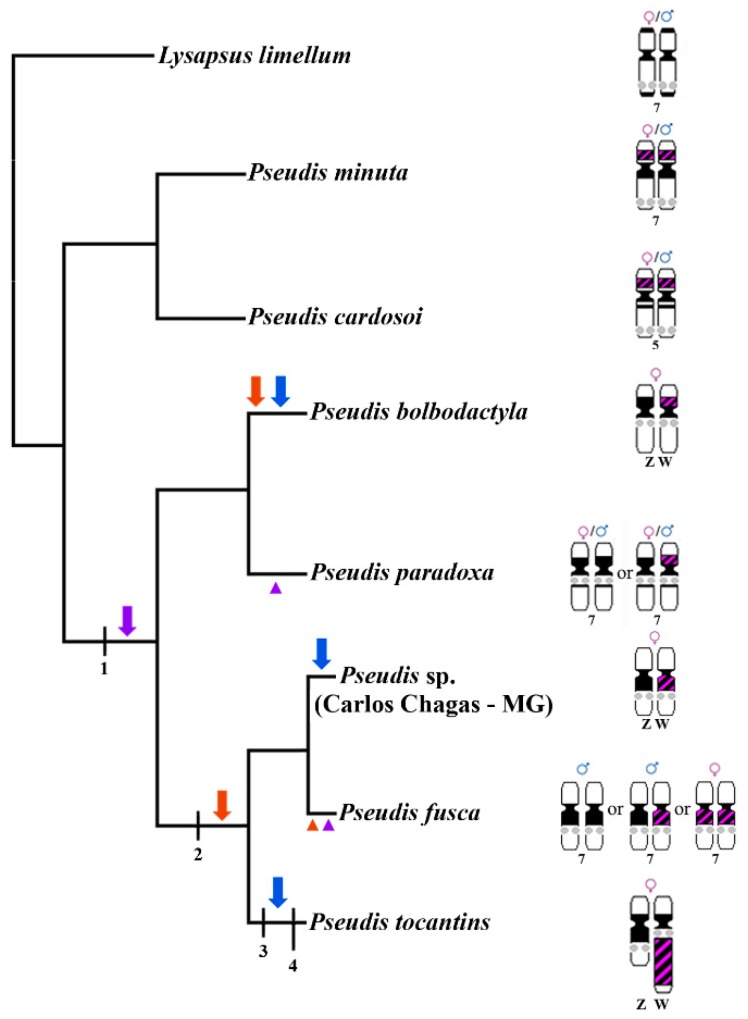
Evolutionary hypothesis for sex chromosome evolution in *Pseudis*. The cladogram shows the phylogenetic relationships inferred in the present work and the ideograms of the NOR-bearing chromosomes. In the ideograms, the regions of PcP190 satDNA are shown in pink, heterochromatic blocks in black, and NOR in gray circles. Ideograms are based on data from Busin et al. [9,48], Gatto et al. [10,11], and the present study. Character changes in the NOR-bearing chromosomes of *Pseudis*: (1) paracentromeric inversion that moved the NOR from a distal region to a region closer to the centromere; (2) pericentromeric inversion that moved the PcP190 site from the short arm to the long arm; (3) paracentromeric inversion that moved the NOR to a pericentromeric area and the PcP190 site to the interstitial region in the W chromosome of *P. tocantins*; and (4) amplification of heterochromatin in the W chromosome of *P. tocantins*. Blue, orange, and purple arrows or arrowheads represent three alternative hypotheses for the sex-linked heteromorphism observed in *P. tocantins*, *Pseudis* sp., and *P. bolbodactyla*. Arrows indicate acquisition of the sex chromosome heteromorphism related to PcP190 satDNA. Arrowheads indicate loss of sex chromosome heteromorphism related to PcP190 satDNA.

Analysis of the cytogenetic data obtained previously in light of the phylogenetic inferences presented here does not discriminate between three equally parsimonious hypotheses: (i) PcP190 satDNA became sex-related in the common ancestor of *Pseudis bolbodactyla*, *P. fusca*, *Pseudis* sp., *P. paradoxa*, and *P. tocantins*, and this condition was lost in *P. fusca* and *P. paradoxa*; (ii) sex heteromorphism of PcP190 satDNA arose independently in the lineage that gave rise to *P. bolbodactyla* and in the common ancestor of *P. tocantins*, *P. fusca*, and *Pseudis* sp., and this condition was lost in *P. fusca*; and (iii) the cluster of PcP190 satDNA emerged as a sex-linked character independently in *P. bolbodactyla*, *Pseudis* sp., and *P. tocantins* (Figure 8). In contrast, in the present study, we may infer that the pericentromeric inversion assumed to carry the PcP190 site from the short arm to the long arm of the NOR-bearing chromosome during the evolutionary history of *Pseudis* [11] occurred in the common ancestor of *P. fusca*, *Pseudis* sp., and *P. tocantins* (Figure 8). In the previous study that proposed the occurrence of this pericentromeric inversion in *Pseudis* [11], the authors could not discard the hypothesis that such an inversion had occurred in the lineage that gave rise exclusively to *P. tocantins*, since no information was available for *P. fusca*. Because we revealed here that *P. fusca* and *Pseudis* sp. share with *P. tocantins* the positioning of a PcP190 cluster in the long arm of chromosome 7, differing from the remaining species of *Pseudis*, the hypothesis suggesting the occurrence of a pericentromeric event has emerged as the most likely option.

In addition to the abovementioned pericentromeric inversion, the W chromosome of *Pseudis tocantins* shows evidence of a paracentromeric inversion that moved the NOR to a pericentromeric region [9,11]. The occurrence of this paracentromeric inversion may be related to the conspicuous accumulation or amplification of repetitive sequences (such as PcP190 satDNA) in the W chromosome of *P. tocantins*. Chromosome inversion events are considered primordial steps of sex chromosome differentiation because they create a situation in which recombination suppression in the sex-specific region is positively selected (review in [1,3,4]). Concomitantly, repetitive DNA accumulation occurs because of the recombination suppression between Z/X and W/Y [3].

Interestingly, even in other lineages with sex-linked PcP190 clusters (*Pseudis bolbodactyla*, and probably, *Pseudis* sp.), no evidence of expressive accumulation/amplification of PcP190 sequences in the W chromosome is present. Therefore, we may suggest that the paracentromeric inversion that occurred in the W chromosome of *P. tocantins* could have favored heterochromatin accumulation in this case because it may have contributed to recombination suppression between the Z and W chromosomes in this species. Based on these assumptions, it is expected that other satDNA sequences, as well as transposable elements, could also have accumulated in the W chromosome of *P. tocantins*. This situation was already reported for several other organisms, such as *Drosophila miranda* [50], the fish *Cynoglossus semilaevis* [51], and the model plant species *Rumex acetosa* [52]. Consequently, the identification of putative transposable elements and other satDNA accumulation in the W chromosome of *P. tocantins* could help further investigation of the sex chromosomes of this species.

In addition, we may hypothesize that only a small differential accumulation of PcP190 satDNA in the sex chromosomes of *Pseudis* may not be sufficient for the maintenance of recombination suppression between their pericentromeric heterochromatic blocks. This hypothesis is raised from the mapping of PcP190 in *P. fusca* and *P. paradoxa*, which indicates the existence of polymorphic conditions at PcP190 sites that are not related to sex. The situation observed in *P. fusca* and *P. paradoxa* may represent the loss of sex-related condition (alternatives indicated as arrowheads in Figure 8), possibly achieved by recombination between the PcP190-positive pericentromeric block of ancestral W chromosomes and the PcP190-negative pericentromeric block of ancestral Z chromosomes. Occasional recombination events between sex chromosomes have been demonstrated to occur in anurans, representing one explanation for the prevalence of homomorphic sex chromosomes (i.e., fountain of youth model) among frogs [53,54,55]. On the other hand, recombination events and consequently the homogenization of previous sex chromosomes may occur after a transition in the sex determination system, which results in sex chromosome turnover [56]. Sex determination transitions are also a known mechanism that explain the high proportion of homomorphic sex chromosomes among frogs [55,56]. However, to assess if one of these mechanisms resulted in the putative loss of sex chromosome heteromorphism in *P. fusca* and *P. paradoxa*, or even if the condition present in these species is plesiomorphic (assumption considered in the alternative indicated by blue arrows in Figure 8), more studies are still necessary.

## Figures and Tables

**Table 1:** Genetic distances (%) based on a 482 bp segment of the 16S mitochondrial gene (16Sar-16Sbr fragment—below the diagonal) and the H1 fragment (above the diagonal) between the species and populations of *Pseudis*. Note the high values of genetic distance (bold) found between *Pseudis fusca* from Carlos Chagas–MG and the other *Pseudis fusca* populations. In the diagonal, in gray, intraspecific genetic distances (%) based on the 16Sar-16Sbr fragment of the 16S rRNA gene (left) or H1 fragment (right). CM: Coronel Murta-MG, Brazil. Sal: Salinas-MG, Brazil. Ara: Araçuaí-MG, Brazil. Jeq: Jequitinhonha-MG, Brazil.

	1	2	3	4	5	6	7
1. *P. fusca* (CM/Sal/Ara/Jeq)	0/0.13	5.98 *	7.77	8.71	8.81	13.59	13.53
2. *P. fusca* (Carlos Chagas)	7.16	0/0.17	6.30	7.79	7.82	12.64	12.85
3. *P. tocantins*	6.99	6.08	0.42/0.45	8.45	8.70	13.64	13.77
4. *P. bolbodactyla*	8.02	8.15	7.05	2.57/2.30	5.66	12.24	12.39
5. *P. paradoxa*	8.88	9.12	8.15	6.07	2.40/2.73	12.40	12.48
6. *P. minuta*	13.64	11.82	12.55	11.83	13.01	0.20/0.62	1.47
7. *P. cardosoi*	13.06	11.97	12.65	11.62	12.72	1.25	0.41/0.39

* Not including sequences from the Jequitinhonha, state of Minas Gerais, population, for which only the 16Sar-16Sbr fragment was available.

## Supplementary Materials

The following are available online at http://www.mdpi.com/2073-4425/10/2/160/s1, Table S1: Specimens of *Pseudis fusca* and *Pseudis* sp. used for amplification of PcP190 nucleotide sequences by polymerase chain reaction (PCR), Southern blotting (SB), and fluorescent in situ hybridization (FISH). Table S2: Mitochondrial sequences obtained from GenBank and generated in the present work. Figure S1: Alignment of the PcP190 satellite DNA sequences isolated from *Pseudis fusca* with all PcP-2 sequences described to date (with completely or incompletely sequenced CR), and all PcP190 sequences from the remaining groups that present completely sequenced CR.
