# Supplementary material for: Satellite DNA Mapping in Pseudis fusca (Hylidae, Pseudinae) Provides New Insights into Sex Chromosome Evolution in Paradoxical Frogs
Source: Genes (Basel). 2019 Feb 19;10(2):160. doi: 10.3390/genes10020160 (PMC6410007; doi:10.3390/genes10020160)
Supplement: Supplementary file 1 [file genes-10-00160-s001.pdf]

Table S1. Specimens of *Pseudis fusca* used in this work. The specimens used for isolation of PcP190 nucleotide sequences by PCR (PCR), Southern blotting (SB) and fluorescent *in situ* hybridization (FISH). In addition, the chromosome locations of the PcP190 sites are indicated by the identification of the chromosome arms that bear those sites (7q: long arm of chromosome 7; Wq: long arm of chromosome W). Heterozygous and homozygous conditions are also presented. ZUEC: Museu de Zoologia "Prof. Adão José Cardoso", Universidade Estadual de Campinas, Campinas-SP, Brazil. MNRJ: Museu Nacional, Rio de Janeiro, State of Rio de Janeiro(RJ), Brazil. UFMG-A: Coleção de Anfíbios da Universidade Federal de Minas Gerais, Belo Horizonte, State of Minas gerais (MG), Brazil.

Species/Specimen voucher	Sex	Specimen locality	PCR	SB	FISH
ZUEC 13235	male	Coronel Murta/state of MG	X	ladder pattern	7q
ZUEC 13236	male	Coronel Murta/ state of MG		no signal	
ZUEC 13237	male	Coronel Murta/ state of MG		no signal	
ZUEC 13238	male	Coronel Murta/ state of MG		no signal	
ZUEC 13239	male	Coronel Murta/ state of MG		no signal	
MNRJ 35459	male	Coronel Murta/ state of MG		no signal	
MNRJ 35460	male	Coronel Murta/ state of MG			7q
MNRJ 35461	male	Coronel Murta/ state of MG		no signal	no signal
ZUEC 24517	male	Salinas/ state of MG		no signal	no signal
ZUEC 24518	female	Salinas/ state of MG	X	ladder pattern	7q/7q
ZUEC 22076	female	Carlos Chagas/ state of MG		ladder pattern	
ZUEC 22077	female	Carlos Chagas/ state of MG		ladder pattern	Wq
UFMG-A 17455	female	Carlos Chagas/ state of MG		ladder pattern	
UFMG-A 17457	female	Carlos Chagas/ state of MG		ladder pattern	
UFMG-A 17468	female	Carlos Chagas/ state of MG		ladder pattern	
UFMG-A 15136	female	Carlos Chagas/ state of MG		ladder pattern	
UFMG-A 17469	male	Carlos Chagas/ state of MG		no signal	
ZUEC 22078	male	Carlos Chagas/ state of MG		no signal	no signal
ZUEC 22079	male	Carlos Chagas/ state of MG		no signal	

Table S2. Mitochondrial sequences obtained from GenBank and generated in the present work.

Taxon	Museum Voucher	GenBank Accession Numbers (H1/cytb)	Locality
Lysapsus bolivianus	MNRJ 33883	MK293746/MK322486[4]	Santarém, state of Pará, BR
Lysapsus bolivianus	MNRJ 33878	MK293745/MK322485[4]	Santarém, state of Pará, BR
Lysapsus bolivianus	MNRJ 33954	MK293752/MK322492[4]	Guajará-Mirim, state of Rondônia, BR
Lysapsus bolivianus	MNRJ 33944	MK293750/MK322490[4]	Guajará-Mirim, state of Rondônia, BR
Lysapsus bolivianus	MNRJ 33972	MK293751/MK322491[4]	Guajará-Mirim, state of Rondônia, BR
Lysapsus caraya	ZUEC 13216	MK293754/MK322494[4]	Santa Terezinha, state of Mato Grosso, BR
Lysapsus caraya	ZUEC 13204	MK293753/MK322493[4]	Santa Terezinha, state of Mato Grosso, BR
Lysapsus laevis	AM-CC 101720	AY843696/AY843941	Aishalton, GUY
Lysapsus laevis	FN 011[5]	MK293755/MK322495[4]	Pacaraima, state of Roraima, BR
Lysapsus laevis	FN 012[5]	MK293756/MK322496[4]	Pacaraima, state of Roraima, BR
Lysapsus limellum	MACN 38645	AY843697/AY843942	Corrientes, ARG
Lysapsus limellum	MNRJ 34067	MK293744/MK322484[4]	Corumbá, state of Mato Grosso do Sul, BR
Lysapsus limellum	MNRJ 34079	MK293747/MK322487[4]	Nossa Senhora do Livramento, state of Mato Grosso, BR
Lysapsus limellum	MNRJ 34071	MK293748/MK322488[4]	Nossa Senhora do Livramento, state of Mato Grosso, BR
Lysapsus limellum	MNRJ 34076	MK293749/MK322489[4]	Nossa Senhora do Livramento, state of Mato Grosso, BR
Lysapsus limellum	MNRJ 34060	MK293743/MK322483[4]	Corumbá, state of Mato Grosso do Sul, BR
Pseudis bolbodactyla	CHUNB 42764	EF153005	Aporé, state of Goiás, BR
Pseudis bolbodactyla	CHUNB 42879	EF153007	Pirapora, state of Minas Gerais, BR
Pseudis bolbodactyla	CHUNB 42658	EF153006	Alvorada do Norte, state of Goiás, BR
Pseudis bolbodactyla	MNRJ 34041	MK293734/MK322474[4]	Quirinópolis, state of Goiás, BR
Pseudis cardosoi	CHUNB 42610	EF152997	Jaquirana, state of Rio Grande do Sul, BR
Pseudis cardosoi	ZUEC 11601	MK293732/MK322472[4]	Tainhas, state of Rio Grande do Sul, BR
Pseudis cardosoi	CAUPF 1525	MK293733/MK322473[4]	São Francisco de Paula, state of Rio Grande do Sul, BR

Pseudis fusca	CHUNB 42625	EF153003	Araçuaí, state of Minas Gerais, BR
Pseudis fusca	ZUEC 13236	MK293741/MK322481[4]	Coronel Murta, state of Minas Gerais, BR
Pseudis fusca	MNRJ 35459	MK293742/MK322482[4]	Coronel Murta, state of Minas Gerais, BR
Pseudis fusca[1]	ZUEC 13235	MH632121/MK322480[4]	Coronel Murta, state of Minas Gerais, BR
Pseudis fusca[1]	ZUEC 24518	MH632122/MK293761	Salinas, state of Minas Gerais, BR
Pseudis fusca[2]	MTR_ALCX195P5	KU495489	Jequitinhonha, state of Minas Gerais, BR
Pseudis fusca[2]	MTR_ALCX197P6	KU495490	Jequitinhonha, state of Minas Gerais, BR
Pseudis fusca[1]	ZUEC 22077	MH632123/MK293759	Carlos Chagas, state of Minas Gerais, BR
Pseudis fusca[1]	ZUEC 22078	MH632124/MK293760	Carlos Chagas, state of Minas Gerais, BR
Pseudis minuta	CHUNB 34687	EF152996	Porto Alegre, state of Rio Grande do Sul, BR
Pseudis minuta	MACN 37786	AY843739/AY843985	Entre Ríos, ARG
Pseudis paradoxa	CHUNB 42928	EF153009	Tartarugalzinho, state of Amapá, BR
Pseudis paradoxa	CHUNB 43032	EF153010	Pinheiro, state of Maranhão, BR
Pseudis paradoxa	CHUNB 43002	EF153011	Boa Vista, state of Roraima, BR
Pseudis paradoxa	UTA 53104	EF153012	Mabaruma, GUY
Pseudis paradoxa	MACN 38584	AY549364	Formosa, ARG
Pseudis paradoxa	DCC3284	AY364541	Brazil
Pseudis paradoxa	MACN 38642	AY843740/AY549417	Corrientes, ARG
Pseudis paradoxa	CHUNB 42848	EF153008	Corumbá, state of Mato Grosso do Sul, BR
Pseudis paradoxa	DCC3284	AY326032	Luiz Antônio, state of São Paulo, BR
Pseudis paradoxa	ZUEC 12828	MK293735/MK322475[4]	Nova Itapirema, state of São Paulo, BR
Pseudis paradoxa	ZUEC 12829	MK293739/MK322479[4]	Bacabal, state of Maranhão, BR
Pseudis paradoxa	MNRJ 33859	MK293738/MK322478[4]	Bacabal, state of Maranhão, BR
Pseudis paradoxa	FN 189[5]	MK293737/MK322477[4]	Macapá, state of Amapá, BR
Pseudis paradoxa	FN 013[5]	MK293736/MK322476[4]	Pacaraíma, state of Roraima, BR
Pseudis tocantins	CHUNB 42943	EF153004	Sandolândia, state of Tocantins, BR
Pseudis tocantins	ZUEC 13227	MK293757/MK322497[4]	Porto Nacional, state of Tocantins, BR
Pseudis tocantins	ZUEC 13229	MK293758/MK322498[4]	Porto Nacional, state of Tocantins, BR
Pseudis tocantins	MNRJ 35457	MK293740/MK322499[4]	Porto Nacional, state of Tocantins, BR
Pseudis tocantins[3]	ZUEC 22352	MH571152	Porto Nacional, state of Tocantins, BR

[1] Sequences generated in the present work. [2] Sequences with 521 bp used only in the genetic distance analysis. [3] Sequences retrieved from the mitochondrial genome of *P. tocantins* [1]. [4] Sequences from Aguiar-Júnior et al. [2]. [5] Field number of Ulysses Caramaschi (to be accessioned in the MNRJ collection).

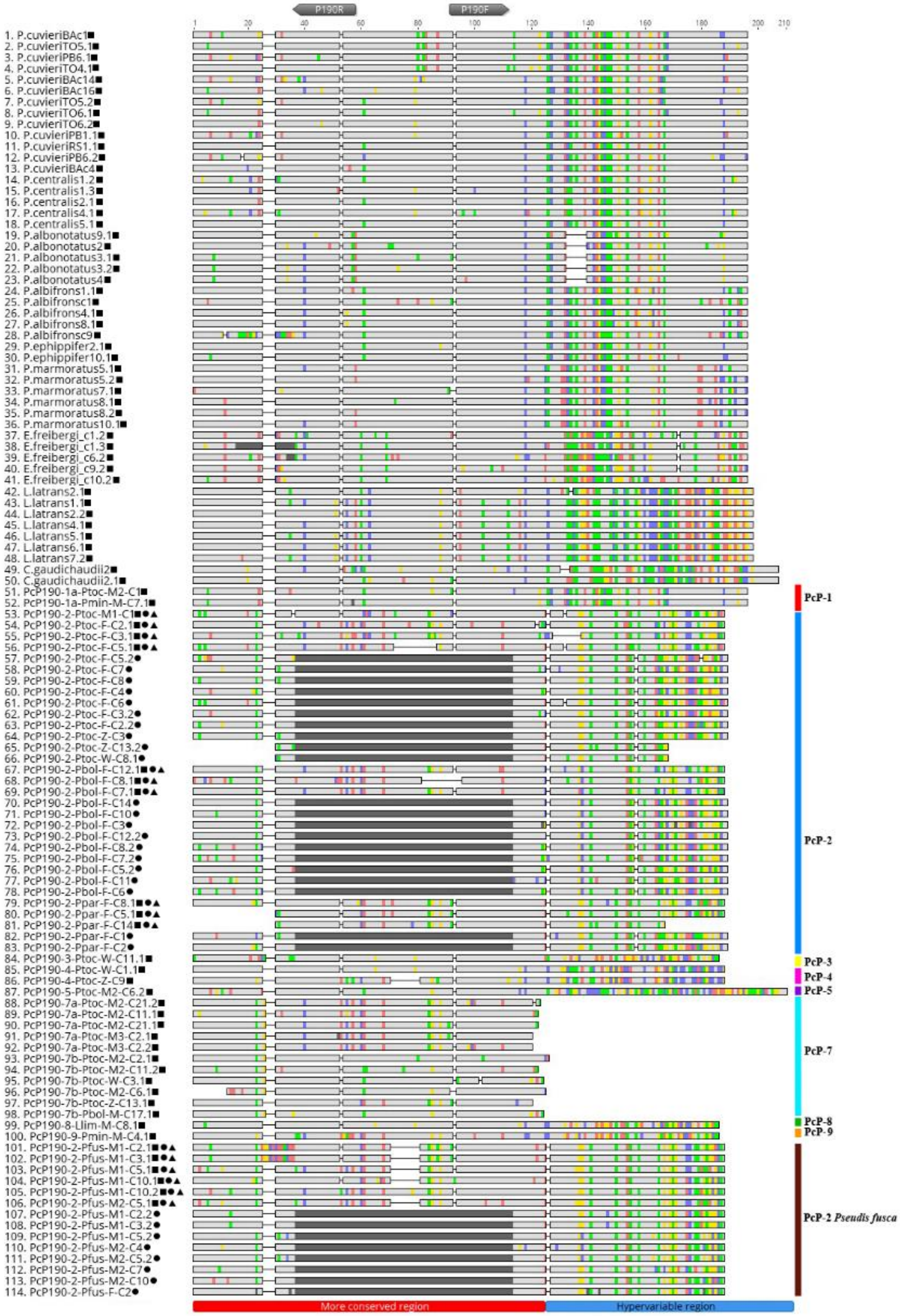
P190R
P190F
1 20 40 60 80 100 120 140 160 180 200 210
1. P.cuvieriBAc1■
2. P.cuvieriTO5.1■
3. P.cuvieriPB6.1■
4. P.cuvieriTO4.1■
5. P.cuvieriBAc14■
6. P.cuvieriBAc16■
7. P.cuvieriTO5.2■
8. P.cuvieriTO6.1■
9. P.cuvieriTO6.2■
10. P.cuvieriPB1.1■
11. P.cuvieriRS1.1■
12. P.cuvieriPB6.2■
13. P.cuvieriBAc4■
14. P.centralis1.2■
15. P.centralis1.3■
16. P.centralis2.1■
17. P.centralis4.1■
18. P.centralis5.1■
19. P.albonotatus9.1■
20. P.albonotatus2■
21. P.albonotatus3.1■
22. P.albonotatus3.2■
23. P.albonotatus4■
24. P.albifrons1.1■
25. P.albifronsc1■
26. P.albifrons4.1■
27. P.albifrons8.1■
28. P.albifronsc9■
29. P.ephippifer2.1■
30. P.ephippifer10.1■
31. P.marmoratus5.1■
32. P.marmoratus5.2■
33. P.marmoratus7.1■
34. P.marmoratus8.1■
35. P.marmoratus8.2■
36. P.marmoratus10.1■
37. E.freibergi_c1.2■
38. E.freibergi_c1.3■
39. E.freibergi_c6.2■
40. E.freibergi_c9.2■
41. E.freibergi_c10.2■
42. L.latrans2.1■
43. L.latrans1.1■
44. L.latrans2.2■
45. L.latrans4.1■
46. L.latrans5.1■
47. L.latrans6.1■
48. L.latrans7.2■
49. C.gaudichaudii2■
50. C.gaudichaudii2.1■
51. PcP190-1a-Ptoc-M2-C1■
52. PcP190-1a-Pmin-M-C7.1■
53. PcP190-2-Ptoc-M1-C1■●▲
54. PcP190-2-Ptoc-F-C2.1■●▲
55. PcP190-2-Ptoc-F-C3.1■●▲
56. PcP190-2-Ptoc-F-C5.1■●▲
57. PcP190-2-Ptoc-F-C5.2●
58. PcP190-2-Ptoc-F-C7●
59. PcP190-2-Ptoc-F-C8●
60. PcP190-2-Ptoc-F-C4●
61. PcP190-2-Ptoc-F-C6●
62. PcP190-2-Ptoc-F-C3.2●
63. PcP190-2-Ptoc-F-C2.2●
64. PcP190-2-Ptoc-Z-C3●
65. PcP190-2-Ptoc-Z-C13.2●
66. PcP190-2-Ptoc-W-C8.1●
67. PcP190-2-Pbol-F-C12.1■●▲
68. PcP190-2-Pbol-F-C8.1■●▲
69. PcP190-2-Pbol-F-C7.1■●▲
70. PcP190-2-Pbol-F-C14●
71. PcP190-2-Pbol-F-C10●
72. PcP190-2-Pbol-F-C3●
73. PcP190-2-Pbol-F-C12.2●
74. PcP190-2-Pbol-F-C8.2●
75. PcP190-2-Pbol-F-C7.2●
76. PcP190-2-Pbol-F-C5.2●
77. PcP190-2-Pbol-F-C11●
78. PcP190-2-Pbol-F-C6●
79. PcP190-2-Ppar-F-C8.1■●▲
80. PcP190-2-Ppar-F-C5.1■●▲
81. PcP190-2-Ppar-F-C14■●▲
82. PcP190-2-Ppar-F-C1●
83. PcP190-2-Ppar-F-C2●
84. PcP190-3-Ptoc-W-C11.1■
85. PcP190-4-Ptoc-W-C1.1■
86. PcP190-4-Ptoc-Z-C9■
87. PcP190-5-Ptoc-M2-C6.2■
88. PcP190-7a-Ptoc-M2-C21.2■
89. PcP190-7a-Ptoc-M2-C11.1■
90. PcP190-7a-Ptoc-M2-C21.1■
91. PcP190-7a-Ptoc-M3-C2.1■
92. PcP190-7a-Ptoc-M3-C2.2■
93. PcP190-7b-Ptoc-M2-C2.1■
94. PcP190-7b-Ptoc-M2-C11.2■
95. PcP190-7b-Ptoc-W-C3.1■
96. PcP190-7b-Ptoc-M2-C6.1■
97. PcP190-7b-Ptoc-Z-C13.1■
98. PcP190-7b-Pbol-M-C17.1■
99. PcP190-8-Llim-M-C8.1■
100. PcP190-9-Pmin-M-C4.1■
101. PcP190-2-Pfus-M1-C2.1■●▲
102. PcP190-2-Pfus-M1-C3.1■●▲
103. PcP190-2-Pfus-M1-C5.1■●▲
104. PcP190-2-Pfus-M1-C10.1■●▲
105. PcP190-2-Pfus-M1-C10.2■●▲
106. PcP190-2-Pfus-M2-C5.1■●▲
107. PcP190-2-Pfus-M1-C2.2●
108. PcP190-2-Pfus-M1-C3.2●
109. PcP190-2-Pfus-M1-C5.2●
110. PcP190-2-Pfus-M2-C4●
111. PcP190-2-Pfus-M2-C5.2●
112. PcP190-2-Pfus-M2-C7●
113. PcP190-2-Pfus-M2-C10●
114. PcP190-2-Pfus-F-C2●
PcP-1
PcP-2
PcP-3
PcP-4
PcP-5
PcP-7
PcP-8
PcP-9
PcP-2 *Pseudis fusca*
More conserved region
Hypervariable region

Figure S1. Alignment of the PcP190 satellite DNA sequences isolated from *Pseudis fusca* with all PcP-2 sequences described to date (with completely or incompletely sequenced CR) and all PcP190 sequences from the remaining groups that present completely sequenced CR. The species whose sequences are shown and the GenBank accession numbers of those PcP190 sequences are as follows: *Physalaemus albifrons* (KM361694 - KM361698), *Ph. albonotatus* (KM361689 - KM361693), *Ph. cuvieri* (JF281109 - JF281125 and KM361673 - KM361683), *Ph. centralis* (KM361684 - KM361688), *Ph. ephippifer* (KM361699 and KM361700), *Ph. marmoratus* (KM361701 - KM361706), *Engystomops freibergi* (MK491531 - MK491535), *Leptodactylus latrans* (KM361718 - KM361724), *Crossodactylus gaudichaudii* (KM361725 and KM361726), *Pseudis fusca* (MH571141 – MH571149), *Ps. tocantins* (KX170887 - KX170933), *Ps. bolbodactyla* (MH370388 – MH370396), *Ps. paradoxa* (MH370403 – MH370407), *Ps. minuta* (MH370442) and *Lysapsus limellum* (MH370453). The more conserved region (CR) and hypervariable region (HR) are labeled at the bottom of the figure, and gray pentagons at the top indicate the annealing sites of the primers P190F and P190R. Black squares (■) indicate sequences used for comparing the *P. fusca* sequences with all previously described PcP190 sequences with respect to CR. Black circles (●) indicate the 45 sequences used for comparing the HRs of the PcP-2 sequences. Black triangles (▲) indicate the 16 sequences used for comparison of the CR of the PcP-2 sequence group.

References

1. Gatto, K. P.; Smith, J. J.; Lourenço, L. B. The mitochondrial genome of the endemic Brazilian paradoxical frog *Pseudis tocantins* (Hylidae). *Mitochondrial DNA Part B* **2018**, *3*, 1106-1107, doi: 10.1080/23802359.2018.1508385.
2. Aguiar-Júnior, O.; Bacci-Jr, M.; Lima, A. P.; Rossa-Feres, D. C.; Haddad, C. F. B.; Recco-Pimentel, S. M. Phylogenetic relationships of *Pseudis* and *Lysapsus* (Anura, Hylidae, Hylinae) inferred from mitochondrial and nuclear gene sequences. *Cladistics* **2007**, *23*, 455-463.

© 2019 by the authors. Submitted for possible open access publication under the terms and conditions of the Creative Commons Attribution (CC BY) license (http://creativecommons.org/licenses/by/4.0/).
